# High Throughput Sequencing Reveals Alterations in the Recombination Signatures with Diminishing Spo11 Activity

**DOI:** 10.1371/journal.pgen.1003932

**Published:** 2013-10-31

**Authors:** Beth Rockmill, Philippe Lefrançois, Karen Voelkel-Meiman, Ashwini Oke, G. Shirleen Roeder, Jennifer C. Fung

**Affiliations:** 1 Department of Molecular, Cellular and Developmental Biology, Yale University, New Haven, Connecticut, United States of America; 2 Department of Obstetrics, Gynecology and Reproductive Sciences and Center for Reproductive Sciences, University of California San Francisco, San Francisco, California, United States of America; National Cancer Institute, United States of America

## Abstract

Spo11 is the topoisomerase-like enzyme responsible for the induction of the meiosis-specific double strand breaks (DSBs), which initiates the recombination events responsible for proper chromosome segregation. Nineteen PCR-induced alleles of *SPO11* were identified and characterized genetically and cytologically. Recombination, spore viability and synaptonemal complex (SC) formation were decreased to varying extents in these mutants. Arrest by *ndt80* restored these events in two severe hypomorphic mutants, suggesting that *ndt80*-arrested nuclei are capable of extended DSB activity. While crossing-over, spore viability and synaptonemal complex (SC) formation defects correlated, the extent of such defects was not predictive of the level of heteroallelic gene conversions (prototrophs) exhibited by each mutant. High throughput sequencing of tetrads from *spo11* hypomorphs revealed that gene conversion tracts associated with COs are significantly longer and gene conversion tracts unassociated with COs are significantly shorter than in wild type. By modeling the extent of these tract changes, we could account for the discrepancy in genetic measurements of prototrophy and crossover association. These findings provide an explanation for the unexpectedly low prototroph levels exhibited by *spo11* hypomorphs and have important implications for genetic studies that assume an unbiased recovery of prototrophs, such as measurements of CO homeostasis. Our genetic and physical data support previous observations of DSB-limited meioses, in which COs are disproportionally maintained over NCOs (CO homeostasis).

## Author Summary

Most eukaryotes depend on the meiotic division to segregate each pair of chromosomes properly into their gametes. Chromosome segregation mistakes happening during meiosis are responsible for most miscarriages as well as many diseases such as Down's and Kleinfelter's syndromes in humans. Proper chromosome segregation during meiosis depends on efficient and regulated recombination events that link homologous chromosomes prior to the first meiotic division. These linkages are initiated at double-stranded breaks (DSBs) in chromosomal DNA by Spo11 and associated proteins. We isolated a valuable new set of *SPO11* alleles in yeast with a wide range of Spo11 activity. Genetic analysis and high throughput sequencing of tetrads from these mutants has revealed unexpected features of meiotic recombination. First, Spo11 DSBs likely continue to form throughout a pachytene arrest in cells compromised for Spo11 activity. Second, the number of recombination initiation events in a given meiosis influences the repair outcome of those events. In addition, our results provide support for crossover homeostasis – a phenomenon in which crossovers are disproportionately maintained over other types of repair in the face of a decrease in DSBs.

## Introduction

An important function of meiosis is to precisely segregate one copy of each chromosome into cells that become gametes. This accurate division is accomplished by presenting the meiosis I spindle with homolog pairs that have been linked by crossovers (COs), so that partner chromosomes can segregate from each other. Chromosomes that fail to sustain a CO are at risk of undergoing nondisjunction, and may produce aneuploid gametes that are usually inviable. A single CO can be sufficient for proper segregation. However, those COs that occur too close or too far from the centromere can also be detrimental [1]–[3]. Thus, it is essential for the meiotic cell to control the number and distribution of COs.

Meiotic DSBs formed by Spo11 are regarded as the major initiating events for CO and non-crossover (NCO) recombination in all organisms studied to date [4]. In budding yeast, it has been estimated that 140–220 DSBs result in ∼95 COs per meiosis [5]. Only a broad estimate of how the remaining DSBs are repaired can be made due to limitation in resolution and the current inability to detect certain repair events in absolute amounts. Nevertheless, given the aforementioned limitations, roughly 40 become detectable NCOs [5], [6] and the rest are undetectable events, including restorations (∼40) [7], intersister (∼10–30) and possibly NCOs with very short gene conversion (GC) tracts. Thus the majority of DSBs formed during meiosis are directed to interhomolog repair, in contrast to mitosis where intersister repair dominates.

Efficient interhomolog repair requires that the two homologs pair, at least locally, at DSBs. In yeast and mice, Spo11-initiated recombination has been shown to be essential for chromosomes to find their homologous partners and establish stable pairing [8], [9]. Spo11-initiated recombination is furthermore necessary for SC assembly. Homologous synapsis via SC deposition along the paired chromosomes further biases repair towards a CO outcome [10]. Although a large number of synapsis initiation events are presumed to occur at recombination sites themselves, the earliest synapsis events initiate at the centromeres [11]–[13]. A small set of *spo11* alleles has been previously used to assess the consequences of reduced numbers of DSBs on CO distribution and frequency [14], [15]. These studies revealed the existence of a phenomenon whereby CO numbers remain stable despite fluctuations in total recombination events. It has been proposed that such “CO homeostasis” reflects a mechanism to ensure that COs form at the expense of NCOs when overall DSB levels are low [15].

In budding yeast meiosis, most COs result from resolution of a double Holliday junction, while NCOs arise predominantly through synthesis-dependent strand annealing (SDSA) [7], [16]. Both processes involve intermediates containing heteroduplex DNA. Mismatch repair of the heteroduplex can result in gene conversion that is generally contiguous; however, it is now appreciated that gene conversion tracts may also be discontinuous [6], [7], [17]. Since gene conversions are associated with both COs and NCOs, they have historically been used as a metric for overall recombination. Gene conversion can easily be measured when heteroalleles are used to select prototrophs. In meiosis, repair of the DSB is initiated by basepairing one free end with the non-sister chromatid, forming a heteroduplex. Depending on the length of the gene conversion tract, when one end of the heteroduplex stops between two heteroalleles, a functional gene can be generated, making the cell prototrophic. Prototrophs are a subset of gene conversions that are detectable.

The analysis of flanking marker exchange among prototrophs in the *ARG4* region was used to substantiate the existence of CO homeostasis [15], [18]. Increasingly defective *spo11* alleles generated prototrophs with increasingly higher levels of CO association (i.e., flanking marker exchange in selected prototrophs), suggesting that, as DSBs become scarce, a given recombination event is more likely to be repaired as a CO. A second way to define CO homeostasis was described [19] where the fluctuation of CO number remained low compared to larger fluctuations of total recombination events in wild-type meioses. This alternate analysis refocused the phenomenon of CO control in more physiological ranges of DSBs and paved the way to study CO homeostasis in other organisms [20]. CO homeostasis has now been documented in worm, mouse and human meioses using cytological markers to measure lower than expected fluctuations in CO number [21]–[23].

It is noteworthy that measurement of GC tract lengths is constrained by the density of heterozygous markers. Two studies in yeast [24], [25] measured GC tract lengths, averaging 1–2 kb, among identified recombinants within small well-marked regions. Recently, high density arrays and next-generation sequencing have further refined tract length measurements and expanded the data to encompass the whole genome [5], [6]. CO-associated tract lengths were found to be quite variable, averaging ∼2000 bp, and being ∼200 bp longer than conversion tracts not associated with a reciprocal exchange. Importantly, several recombination mutants have altered GC tract lengths, revealing that mutants can affect recombination in unexpected ways [5], [19].

Since Spo11 is the enzyme that catalyzes DSB formation, the identification of additional alleles can be a valuable tool for deciphering the role of DSB levels in various aspects of meiosis. Here we isolate nineteen new *spo11* alleles and characterize them using genetics and sequencing to evaluate the relationship between recombination initiation and recombination outcomes. Historically, prototroph assays have been used as a genetic measure for DSB levels. We find, that especially for meioses with severely reduced Spo11 activity, prototroph frequencies critically underestimate DSB formation. Our findings indicate that a reduction in DSB activity influences detectability of recombination events due to alterations in the lengths of GC tracts. The extent and direction of tract length change depends on whether the event results in a CO or NCO.

## Results

### Novel *spo11* hypomorphs display a broad spectrum of CO defects

Hypomorphic *spo11* alleles were isolated by screening transformants carrying PCR-derived mutant *SPO11* sequences for defects in gene conversions and spore viability (Materials and Methods). Nineteen non-null mutants were recovered and characterized. The sequence changes in these mutants are presented in Figure S1. Diploids homozygous for each mutation were constructed and used to quantify CO frequencies. Genetic analysis of *spo11* hypomorphs must take into account that mutants with severe defects will generate mostly dead spores due to nondisjunction of nonrecombinant chromosomes [26]. Thus, the spores that are viable may have undergone higher than average numbers of COs. To eliminate this bias, we incorporated a *spo13* mutation in the strains used to make genetic measurements. Meiosis in *spo11 spo13* diploids generates viable diploid spores as a result of a single, predominantly equational round of chromosome segregation, independent of recombination. [27].

**Figure 1:**
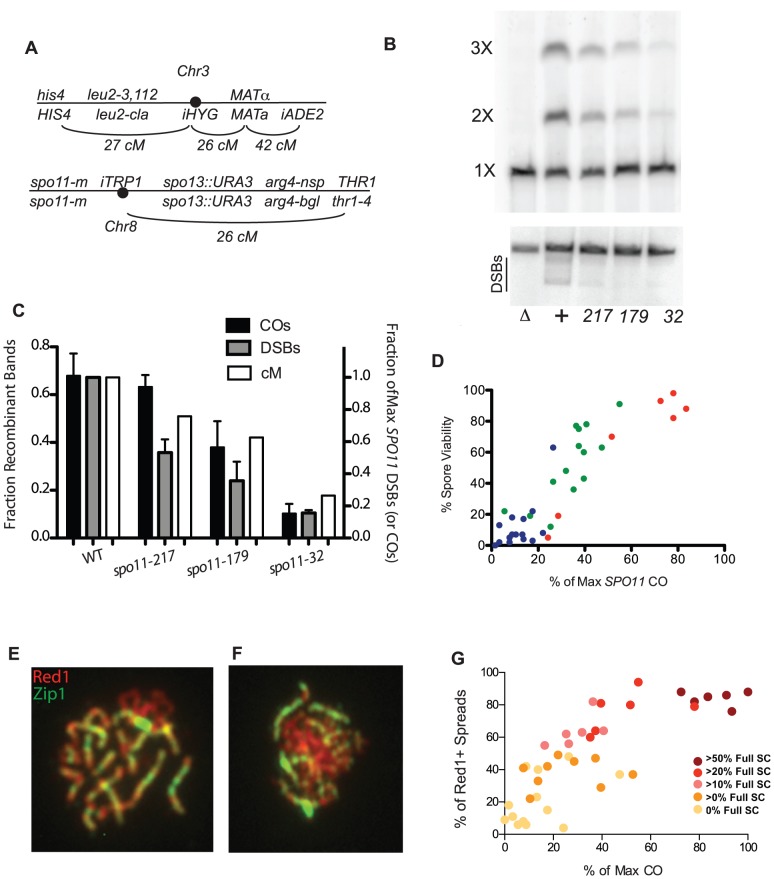
Crossing over, spore viability and chromosome synapsis in *spo11* hypomorphs. A, Marker configuration in diploids. *spo11-m* refers to homozygous *SPO11* alleles. B, Physical analysis of crossing over between linear and circular versions of chromosome 3 and DSB formation on chromosome 3. Top panel: a *spo11* null mutant (Δ),WT (+), and three *spo11* hypomorphs (alleles *217*, *179* and *32*) were sporulated at 31.5°C for 48 hrs. Chromosomes were fractionated on a CHEF gel and examined by Southern blot analysis probing with chromosome 3 sequences. The positions of linear monomers (1×), dimers (2×) and trimers (3×) are indicated. Circular chromosomes do not enter the gel. Bottom panel: Same as top panel, but strains were *sae2Δ*. The smears under the linear chromosome 3 (indicated by the black line) represent the broken circular 3 (just under linear band) and broken linear chromosome 3. C, Quantitation of CO and DSB products. The fraction of Recombinant Bands (dark shaded bars) was calculated as the sum of dimer and twice the trimer band divided by the sum of all three bands. (SDs, black bars). %DSBs (intensity below linear band) were normalized to WT (grey bars) where wild-type cut chromosomes were 18% of the total. Also plotted are the corresponding genetic CO values for each strain (white bars) expressed as a percent of the maximum level of chromosome 3 COs observed (Table S1). D, Spore viability in *spo11* mutants was measured in *SPO13* strains by dissection of at least 20 tetrads (Table S2). The color reflects sporulation temperature (red = 31.5°C, green = 22°C and blue = 18°C). E, F. Examples of spread nuclei (Red1, red and Zip1, green). E: full SC; F: some SC. G. Red1 positive chromosome spreads were scored as “no SC”, “some SC” or “full SC” (Table S2). The percentage of SC containing nuclei, is plotted against CO level, with data points colored to indicate the percentage of SC-containing nuclei showing full synapsis.

A wild-type diploid and twenty mutants (19 novel alleles and a previously characterized allele, *spo11-D290A6HA* [28]) in an isogenic background (Figure 1A) were induced to undergo meiosis at three different temperatures (18°C, 22°C and 31.5°C). To isolate meiotic products for measurements of CO frequencies, arginine prototrophs were selected from purified spore populations. Arg prototrophs result from a GC between heteroalleles at the *ARG4* locus. Arg^+^ recombinants were scored for the segregation of four chromosome 3 markers; CO frequencies on chromosome 3 were then summed to obtain an overall measure of CO frequency (Table S1). The 20 mutants characterized exhibit a wide range of recombination levels. Among the different mutants, crossover defects ranged from 50 fold reduced (for *spo11-751*) to 1.6 fold reduced (for *spo11-118*), at the most restrictive temperature. Interestingly, the vast majority of mutants are cold sensitive; for example, the *spo11-245* mutant exhibits 55% of the wild-type levels of COs at 31.5°C, but only 3.8% of the wild-type level at 18°C.

To obtain an independent measure of crossing over, we conducted a physical analysis of reciprocal recombination between linear and circular versions of chromosome 3 ([29]; Materials and Methods). Wild type (WT) and three *spo11* hypomorphs were sporulated at 31.5°C, and chromosomes from populations of sporulated cells were then fractionated on a CHEF gel and analyzed by Southern blot hybridization using a probe from chromosome 3 (Figure 1B). A single CO between circular and linear chromosomes 3 results in a linear chromosome twice as long as the original. A CO between the linear dimer and the other circle generates a chromosome three times as long as the original. The abundance of chromosome 3 CO products as measured in this assay correlates well with the frequency of COs as determined genetically (Figure 1C) (correlation coefficient = 0.96; p-value<0.05.). DSBs were also physically measured with the same mutant alleles in a *sae2* background and were found to have a reduction in observable DSBs (Figure 1B,C).

### CO levels are correlated with spore viability and SC formation in *SPO11* alleles

Since crossing over is required for proper chromosome segregation at meiosis I, a simple expectation is that spore viability varies with CO frequency in the *spo11* mutants. Spore viability was measured in *SPO13* versions of the *spo11* mutants used to measure crossing over (Table S2). In wild-type yeast, COs are in significant excess to the number of chromosome pairs (∼95 COs vs. 16 pairs of chromosomes). Thus, modest decreases in crossing over are not expected to decrease spore viability dramatically as long as COs are properly distributed among chromosomes [30]. Accordingly, the relationship between CO frequency and spore viability was non-linear; dramatic decreases in viability were observed only as the number of measured COs approached the number of chromosome pairs (Figure 1D). The apparent requirement for more than one CO per chromosome may reflect a defect in crossover control or the inability for a functioning CO distribution system to deal with so few COs. However, we cannot eliminate the possibility that we have a selection bias for high functioning cells within a population due to our method of first selecting prototrophs.

Assembly of the SC begins at the sites of synapsis initiation complexes (SICs) [12], [31] and these complexes are thought to mark the sites of future COs [32], [33]. Thus, the extent of SC formation is presumed to parallel CO level [14]. We characterized *spo11* hypomorphs for the extent of SC formation by staining surface-spread nuclei with antibodies to the SC component, Zip1 (Figures 1E and 1F). The synapsis phenotypes of the mutants encompassed the entire spectrum from mostly dotty Zip1 localization with only rare linear stretches to wild-type levels of full-length SC. Overall, the extent of SC formation in *spo11* mutants roughly follows CO levels with those mutants undergoing more COs achieving more complete synapsis (Figure 1G, Table S2).

### *spo11* hypomorphs can be rescued by prophase arrest

**Figure 2:**
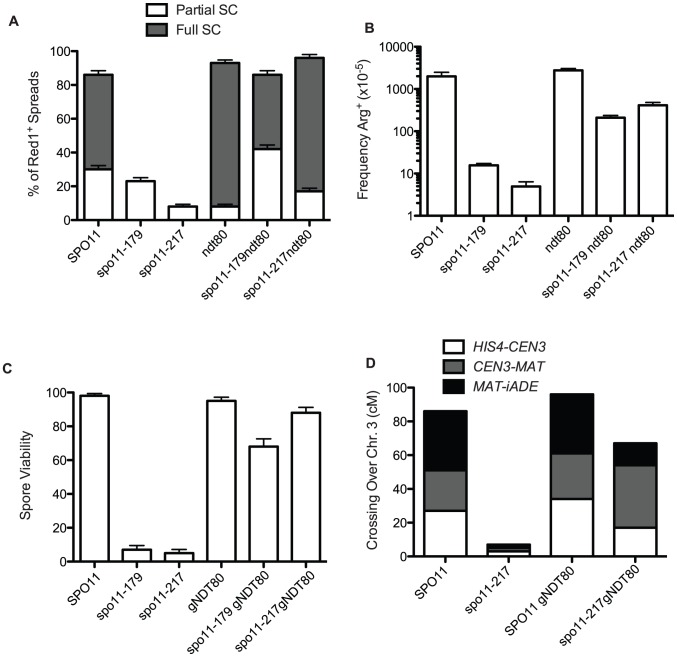
Pachytene arrest rescues *spo11* mutants. A. Extent of SC formed in *spo11* mutants in *NDT80+* and *ndt80* backgrounds after 48 hours sporulation at 18°C. White bars are partial SC (i.e., at least two linear tracts of SC, “some SC” in Table S2) and dark bars represent nuclei with most chromatin containing Zip1 in stretches (“Full SC” in Table S2). At least 100 spreads exhibiting Red1 staining were scored for each mutant. B. Arg prototrophs were measured in “return-to-growth” assays from at least 4 cultures (Table S1) and the average is plotted with standard deviations. C and D. *gNDT80* strains (*pGal-NDT80 – GAL4.ER*) were arrested at pachytene for 48 hours and then induced to sporulate by the addition of estrogen. C. Spore viability was scored among 85 tetrads for *pGal-NDT80*, 172 tetrads for *spo11-217 pGal-NDT80* and 25 tetrads for *spo11-179 pGal-NDT80*. D. At least 100 four-spore-viable tetrads were scored for recombination for three intervals on chromosome 3. Control *NDT80+* data was from tetrad dissection and *spo11-217* data is from isolated *spo13* spores (Table S1, S3).

In order to score synapsis in a uniformly arrested population for several severely defective *spo11* hypomorphs, we measured SC formation in an *ndt80* background. Ndt80 promotes progression beyond the pachytene stage of meiosis, thus *SPO11 ndt80* cells arrest with fully synapsed chromosomes [34]. Interestingly, while some severe *spo11* mutants only produced rare nuclei with partial SCs, in the *ndt80* background such mutants displayed up to 96% of meiotic chromosome spreads with SC (Figure 2A, Table S2). This *ndt80*-associated restoration of synapsis was apparent for several alleles (*179*, *217*, *245*, *1025* and *240*, Table S2 and data not shown) but either weakly restored or absent in other alleles (*246*, *117* and *845*, data not shown). To further examine this apparent rescue of the *spo11* phenotype, recombination levels were measured by scoring Arg prototroph formation after 48 hours of *ndt80*-arrest by a return to growth assay. While previous work reported small increases in heteroallelic prototrophy levels in *ndt80*- and *cdc28-*arrested cells [34], [35] our WT *ndt80* strain did not increase Arg prototrophy levels compared to WT (Figure 2). However, Arg prototroph formation was increased approximately 10 and 60-fold in *spo11-179 ndt80* and *spo11-217 ndt80*, respectively (Figure 2B, Table S1) compared to the mutants in the *NDT80* background, and reached about 10% of WT (and *SPO11 ndt80*) levels.

If recombination and synapsis are restored, then spore viability should increase as well in *spo11* hypomorphs arrested at late pachytene. To explore this question we used an estrogen-inducible *NDT80* allele [36], so that after a prolonged prophase arrest, cells could be induced to sporulate. Dissection of the *spo11* hypomorphs after 48 hours of *ndt80* arrest produced a large increase in spore viability (*spo11-179* increased from 7% to 71% and *spo11-217* increased from 5% to 88%, Figure 2C, Table S2). Moreover, crossing over, increased to about 60% of wild-type values (Figure 2D, Tables S1 and S3).

### *spo11* mutants display more severe defects in heteroallelic recombination than in crossing over

The effect of *spo11* mutations on meiotic GC was assessed by determining the frequency of prototroph formation at *ARG4*. We observed insignificant temperature effects in the wild-type strain for prototroph frequency (2.3×10^−4^ vs. 1.8×10^−4^ when sporulated at 22° or 31.5° respectively). Although CO values appeared to be somewhat temperature sensitive (78 cM vs. 91 cM at 18° and 31.5° respectively, Table S1), no effects of temperature on CO numbers or tract lengths were found by deep sequencing analysis in a limited number of tetrads (see below).

Diaz et al. [28] found that certain *spo11* alleles have altered cut sites within an artificial hotspot, suggesting that alterations of the Spo11 complex can affect more than just quantity of cuts. While we have checked that a subset of our *spo11* alleles have decreased DSBs (Figure 1B) we do not know if they have additional alterations in the locations of cutting. For simplicity of this analysis, we will assume that each allele is a simple hypomorph, and that temperature effects change only the number of events. (Note that in our simulations below, we find that varying the usage of DSB sites within the *ARG4* region does not significantly alter the prototrophy levels (Figure S2)

**Figure 3:**
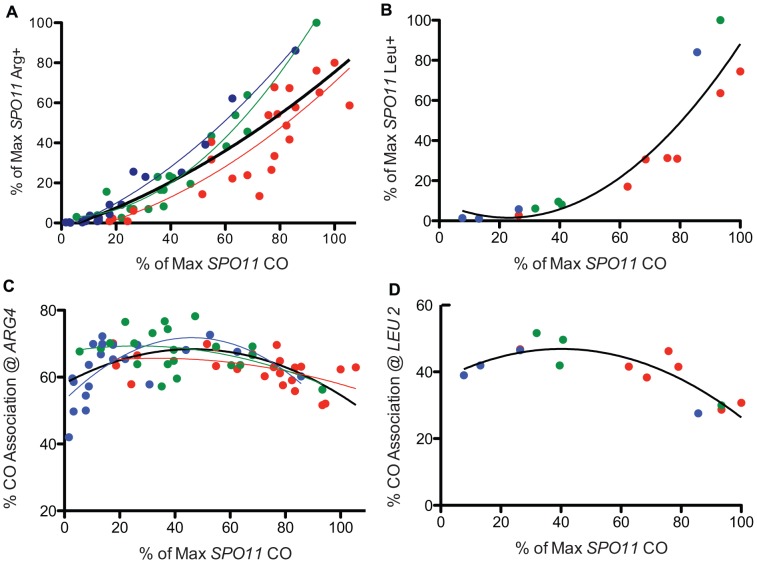
Prototroph formation and CO association in *spo11* hypomorphs. A. WT and *spo11* mutants were sporulated at three temperatures (18°C, 22°C and 31.5°C) and plated to select Arg^+^. Prototroph frequencies, expressed as a percent of the maximum value observed among all strains tested at all temperatures (Table S1), are plotted against overall CO frequencies on chromosome 3, expressed as a percent of the maximum level of *SPO11* COs (Table S1). The color of the data points and the associated least squares fitted lines reflects the temperature in which the diploid was sporulated (red = 31.5°C, green = 22°C and blue = 18°C and the black line encompasses data from all temperatures). B. Prototrophs were measured at *LEU2* for three *spo11* mutants (alleles *217*, *179* and *32*) and WT at three temperatures. Additional points (31.5°C) represent three Spo11 “reduced” mutants (see Table S3, S6). Raw data were normalized as described above and then plotted. The solid black line shows the least squares fit for the complete dataset. C. WT and *spo11* mutants were sporulated at 18°C, 22°C and 31.5°C (blue, green and red data points, respectively) and assayed for flanking marker exchange among Arg prototrophs (Table S1). The colored lines reflect the least squares fit for each temperature and the solid black line represents a least squares fit to the complete dataset. The % CO association @ *ARG4* is the percent of Arg prototrophs with a CO in the *TRP1-THR1* interval. D. Three mutants (alleles *217*, *179* and *32*) and three Spo11 “reduced” mutants (see Table S3, S6) and WT were assayed for CO association at *LEU2*. The percent of Leu prototrophs with a CO in the *HIS4-iHYG@CEN* interval was plotted against overall CO values. The solid black line represents a least squares fit to the data. The formula used for the least squares fit is a second order polynomial.

If prototroph formation and crossing over are similarly impacted by decreased Spo11 activity, then a directly proportional relationship should be observed when CO frequencies are plotted against prototroph frequencies. However, prototroph formation is affected more severely than crossing over (Figure 3A) and this trend is apparent in the entire spectrum of mutants and temperatures. The disparity between crossover and gene conversion levels increased as Spo11 activity declined. For example, a mutant with less than 1% of the wild-type level of prototroph formation exhibits as much as 15% of the wild-type level of crossing over (e.g., *spo11-179* at 18°C, Table S1). This is seen even when mutants with greater than 20% of wild-type CO levels are plotted by temperature i.e. the three regression lines would have a positive X intercept rather than at zero. The use of *spo13* dyads to generate spores avoids a viability bias in these analyses. However, if there is significant heterogeneity on a cell-to-cell basis for recombination within a genotype, it is possible that the observed disparity of CO and prototroph frequencies may be somewhat exaggerated. A similar trend to Arg prototrophy was observed when prototroph formation was measured at the *LEU2* locus on chromosome *3*, using a subset of *SPO11* hypomorphs (Figure 3B, Table S4).

To better understand the relationship between GC and crossing over, we examined Arg prototrophs for the frequency of crossing over between flanking markers. In WT, the fraction of Arg prototrophs associated with a CO was ∼59% (Table S1). In the mutants, the fraction of Arg prototrophs associated with a CO was about 1.4 fold higher than in WT (Figure 3C), reaching a maximum of 78% crossover association, at the point where overall CO frequency (relative CO frequency on chromosome 3) decreased to ∼50% of the wild-type level (*spo11-845* at 22°C, Table S1). However, when overall CO values decreased below 50%, the fraction of Arg prototrophs associated with a CO fell with the severity of the allele. A similar trend was observed when conversion-associated COs were measured at *LEU2* (Figure 3D, Table S4). Since the majority of the mutants are cold-sensitive, the bulk of the data providing the downward trend in CO association (i.e., the most severe alleles) were derived from sporulation at 18°C. Thus, it is possible that this may reflect a temperature effect on CO association rather than a result of reduced Spo11 activity. However, inspection of the few alleles that provide similar Spo11 activity at high and low temperatures do not support the notion that cold temperatures influence CO association outcomes (Table S1, S2). With this caveat, we analyze the data using the simpler model that Spo11 activity is responsible for changes in CO association.

Our genetic experiments reveal two unexpected results. First, heteroallelic gene conversion levels (measured at *ARG4* and *LEU2*) fell at a much greater rate especially in alleles with severely compromised Spo11 activity than other indicators of recombination ability (e.g. CO levels, spore viability and SC formation). Second, although flanking marker exchange among Arg and Leu prototrophs increased as expected in *spo11* alleles with moderate defects (presumably due to CO homeostasis), in the most severe alleles, CO association surprisingly decreased compared to less severe mutants. Previous work using moderate *spo11* alleles noticed a weakening of CO homeostasis as DSB activity decreased and proposed that CO homeostasis was strongest in cells with relatively high levels of DSBs [15]. If the trend of CO homeostasis had continued, we would expect that the alleles where Spo11 activity is severely compromised would show a continued gradual increase in CO/NCO ratio. Together, the hyper-reduction of prototrophs and the decrease in CO association suggests that the effects of severely reduced DSBs results not only in fewer events, but influences their repair outcome.

### Whole-genome sequencing suggests a change in the CO/NCO ratio in *spo11* hypomorphs

To better understand the effects of *spo11* mutations on meiotic gene conversion and crossing over, we monitored meiotic recombination on a genome-wide scale by sequencing the genomes of spores from tetrads containing four viable spores. For this analysis, we used a diploid derived by mating a typical laboratory strain (S96) to a clinical isolate (YJM789). The parental strains differ from each other at ∼55,000 locations (single-nucleotide polymorphisms (SNPs)) allowing most interhomolog recombination events to be detected [6], [37].

Spores from five tetrads derived from *spo11* hypomorphs (three from *spo11-217* and two from *spo11-32*, sporulated at 24 or 31.5 ^o^C) were subjected to high-throughput, multiplexed sequencing. The data were compared to sequenced data from three wild-type tetrads sporulated at 30°C of which two were reported previously [6]; the wild-type tetrads had an average of 92 COs (Table 1). We also analyzed one wild-type tetrad at 22°C and two wild-type tetrads at 26°C. The recombination characteristics of the wild-type tetrads do not differ with temperature (Table S5). Two of the tetrads from the *spo11-217* mutant had an average of 80 COs (Table 1); these were classified as “high-functioning (HI)”. The remaining tetrad from *spo11-217* and the two tetrads from *spo11-32* sustained an average of 55 COs (Table 1); these were grouped together and classified as “low-functioning (LO)”. Comparison of recombination in these tetrads with the genetic analysis on chromosome 3 reveals a large bias from selecting four-spore viable tetrads from mutants with low spore viability. *spo11-32* strains sporulated at 31.5°C show only 26% of wild-type crossing over in *spo13* dyads compared to 60% of wild-type COs from the two sequenced four-spore viable tetrads. It seems likely that this difference reflects the fluctuation of DSB activity on a cell-by-cell basis and that selecting for four-spore-viable tetrads constrains the range of DSBs in the meioses that we can recover. We assume that the reason a given mutant acts differently in individual tetrads is due to a difference in DSB number, and thus we categorized these tetrads based on numbers of COs (rather than genotype).

**Table 1:** Frequency of event types from sequencing data for three wild-type tetrads and five *spo11* hypomorphs.

Tetrad Genotype	NCO Total # (%)	4∶0 Event	2-Strand NCO	Disc. Event	GC 1 SNP	CO Total # (%)	CO no Tract	NCO on 3^rd^ Strand	4 Strand Dble CO	Disc. Event	GC 1 SNP	GC Total	Total Events
WT29	59 (39%)	1	5	4	9	92 (61%)	23	10	1	9	8	69	151
WT30	40 (30%)	0	4	0	7	94 (70%)	29	6	1	8	12	65	134
WT46	46 (34%)	1	4	1	12	90 (66%)	22	4	0	14	11	68	136
HI-217 31.5°	35 (30%)	0	3	2	1	83 (70%)	26	2	0	6	6	57	118
HI-217 24°	24 (24%)	0	5	0	2	78 (77%)	22	4	1	8	9	55	102
LO-32 31.5°	13 (18%)	0	2	1	0	58 (82%)	16	2	0	8	5	42	71
LO-32 31.5°	18 (25%)	0	2	0	3	54 (75%)	13	3	0	2	4	41	72
LO-217 24°	17 (25%)	0	2	0	3	50 (75%)	15	6	0	8	4	35	67

WT tetrads were sporulated at 30°C. spo11 alleles were sporulated at temperature specified in their name. Columns 2–6 are NCO events and columns 7–13 are CO events. GC is gene conversion. () indicate frequency relative to total detectable events CO + NCO. “4∶0's” are gene conversions where all four chromatids display the same parental configuration. “2-strand NCO” refers to NCOs in which two chromatids exhibit a gene conversion within 3 kb of each other measured end-to-end and are presumably part of the same event. “Disc. Event” is the number of events in which there was discontinuity between multiple gene conversion tracts. GC 1 SNP” refers to the number of GCs in which the tract is only defined by a single marker. “CO - no tracts” are COs without a detectable GC tract. “4 strand dble CO's” are two COs involving all four chromatids and occur within 3 kb of each other. One of these was included in a 2-stranded NCO. “GC total” is the total number of CO events associated with one or more gene conversions. Total Events are Total NCO + Total CO.

Deep sequencing of wild-type tetrads revealed recombination types that included single and multiple COs and NCOs with and without continuous tracts, as well as multi-strand events [6]. Examination of the recombination landscape in the five *SPO11* hypomorphic tetrads revealed a similar proportion of recombination types as observed in WT. The sole exception is a disproportionate reduction of NCOs (Table 1) as will be discussed below. We note that the proportions of multi-strand COs relative to the total COs were found to be the same for *spo11* hypomorphs and WT (Table 1). This suggests that events classified as complex events are likely to arise from a single DSB rather than via multiple DSB events, since reducing the number of DSBs did not decrease the proportion of the multi-strand COs.

CO interference is the nonrandom spacing of COs that is a feature of the normal distribution of COs but not NCOs. Through sequencing analysis we obtained the positions of COs genome-wide and we then examined interference by fitting a gamma distribution to intercrossover distances [19] obtained from grouping all five *spo11* tetrads. This grouping was necessary to obtain sufficient numbers of intercrossover distances. Gamma values obtained from the distribution reflect the strength of CO interference. Consistent with previous genetic analysis of interference that showed no change in interference for *spo11* mutant alleles [15], our genome-wide analysis detected wild-type levels of interference (gamma = 2.0; gamma = 1.0 represents no interference and 1.8 is wild-type interference) [19]. Moreover with the reduction of DSBs, it is likely that some chromosomes will fail to experience a CO. From the five sequenced hypomorphic tetrads we found five instances where small or relatively large chromosomes (chromosomes 1, 8, 8, 13, 14) lacked a crossover (E_0_s). On these 5 E_0_ chromosomes no other events (NCO's) were observed.

Successful detection of NCOs is subject to the density of markers as well as tract length (e.g., short tracts have a reduced probability of converting a marker and thus are less likely to be detected). As shown in Table 1, the total number of detectable events decreased in the mutants, but NCOs decreased to a greater extent than COs. As a result, COs represent a somewhat greater fraction of total events in the mutants (an average of 77.1% in the LO tetrads versus an average of 66.7% in the wild-type tetrads; P = 0.04, two sample t-test), suggesting a relative increase in COs at the expense of NCOs. However, similar to our previous analysis using prototroph formation, this conclusion assumes that the probability of detecting a NCO by sequencing is constant across the spectrum of mutants.

### In *spo11* mutants, GC tract lengths are increased in COs and decreased in NCOs

Gene conversion tracts result from the repair of heteroduplex or double-stranded gaps formed during DSB repair. In order to assess gene conversion tract lengths in *spo11* hypomorphs, we inspected all CO and NCO events for regions of gene conversions, i.e., regions of 3∶1 or 1∶3 segregation of markers flanked by regions of 2∶2 segregation. The distance between the last marker showing 3∶1 segregation and the closest marker showing 2∶2 segregation is highly variable due to the differences in the density of markers within different regions along the chromosomes, leaving the actual tract length ambiguous. For example, a 3∶1 tract that spans only 1 kb may have the next 2∶2 markers at 10 kb on one side and 1 kb on the other. Using the common midpoint method, this tract would be scored as 6.5 kb (1 kb minimum plus half of each maximum tracts) in length. Even a few of these might skew our tract length analysis.

**Figure 4:**
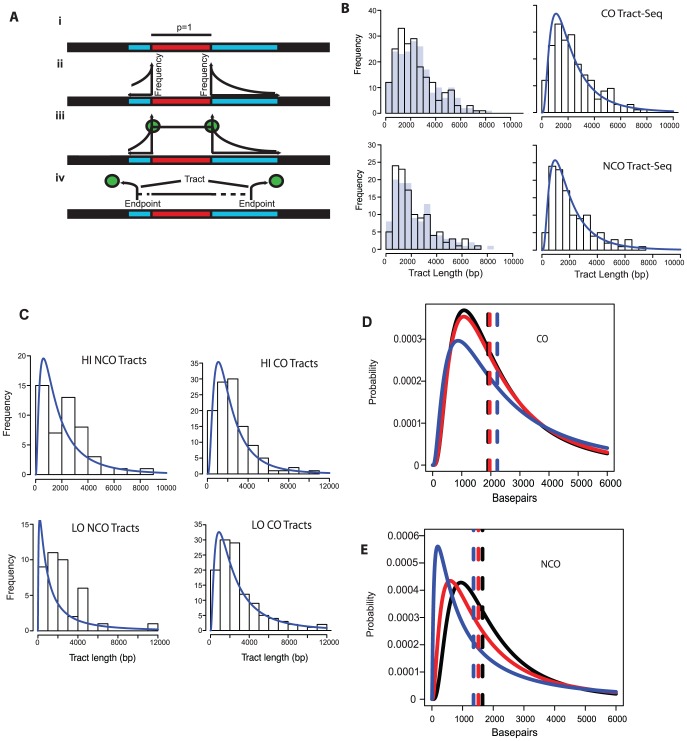
Tract-Seq and summary of sequence data. A. Tract-Seq analysis to generate an estimated tract length, given maximal and minimal tracts. The red rectangle depicts the minimal tract length (“core”) in which reflects a contiguous stretch of 1∶3 markers and blue rectangles on either side of the core represent the regions in which the tract endpoints must be located. (i) The enzymatic resection machinery has processed through the minimal tract. (ii) Assuming a bidirectional enzyme with a symmetric enzymatic processivity, the probability of the enzyme falling off on either side of the core region follows a decreasing exponential law. (iii) Enzymatic complexes (green balls), initially located at the edges of the core region, fall off independently on each side of the core. (iv) The enzymes fall off before reaching the boundaries of the maximal tract. The estimated tract spans the distance between the two endpoints, encompassing the core interval. B. Analysis of wild-type tracts. Left: Histograms of midpoint-estimated (grey bars) and Tract-Seq-estimated (black-outlined transparent bars) individual CO (top) and NCO (bottom) tract lengths. Right: Histograms of Tract-Seq-estimated individual CO (top) and NCO (bottom) tract lengths, with the overlaid best-fitted log-normal distributions of GC tract lengths for each case. C. Histograms of Tract-Seq-estimated (black-outlined transparent bars) of individual NCO (left) and CO (right) tract lengths, along with the overlaid best-fitted log-normal distributions of GC tract lengths for Hi (top) and LO (bottom) mutants. D and E. Tract-Seq was used to estimate tract lengths for GC events associated with COs (D) and for NCOs (E). The probabilities of tracts of different lengths are plotted against tract length (in base pairs). Simulations based on WT (black line), HI (red line) and LO (blue line) tetrads are graphed, and respective means (dotted lines) are shown. Means and 95% confidence intervals are presented in Table 2. A log normal distribution was used to fit the distribution of tract lengths. The mean and standard deviation are the parameters needed to define each log normal distribution.

In order to better define tract lengths, we developed an algorithm, called Tract-Seq, that estimates tract length using a Markov model for tract growth combined with Monte Carlo simulations (Materials and Methods, Figure 4A). Figure 4B shows the distribution of wild-type tract lengths calculated by Tract-Seq in comparison to the distribution calculated by taking the midpoints. We generated similar graphs of “Tract-Seq” lengths for HI and LO (Figure 4C). Average tract lengths were determined from the log-normal fits for the distribution of CO-associated tracts and NCO tracts for WT, HI and LO tetrads (Figure 4D and E). CO-associated tracts increase in length as the Spo11 activity decreases; the means increased by 56 bp for HI tetrads and 316 bp for LO tetrads, compared to WT (Table 2). However, CO-associated tract lengths for LO tetrads, but not HI tetrads, compared to WT are significantly longer (p = 0.05 for LO vs. WT and p = 0.28 for HI vs. WT). In contrast, NCO tracts are smaller in context of decreased Spo11 activity; the mean length of tracts decreased by 134 for HI and 292 bp for LO (Table 2). Again, the difference in NCO tract lengths relative to WT is significant for LO tetrads, but not for HI tetrads (p = 0.02 for LO vs. WT and p = 0.17 for HI vs. WT). For both CO- and NCO-associated tracts, the range of possible tract lengths increases as Spo11 activity decreases.

**Table 2:** Parameters of NCO and CO tracts.

Spo11 Act.	NCO Avg.	NCO 95% CI	CO Avg.	CO 95% CI
WT	1649	382–7124	1906	435–8350
HI	1515	228–10068	1962	427–9010
LO	1357	87–21191	2222	334–14775
30 CO	1180	35–39656	2427	287–20523
10 CO	1037	16–66055	5497	525–57591
3 CO	987	12–78627	14879	1318–168011
NCO 2 Chtd	2436	428–13869		
NCO w/CO	2575	244–27149		

Average lengths in bp of simulated tract lengths for NCO and CO with 95% confidence intervals, which define the variation are listed. “NCO 2 Chtd” are tracts for NCO events with tracts on two chromatids within 3 kb of each other. NCO w/CO are tracts for NCOs within 3 kb of a CO event. Both of these types of events are pooled from all tetrad data.

The decrease in NCO tract length associated with decreasing Spo11 activity makes it possible that the abundance of NCOs relative to COs in the *spo11* mutants was underestimated by sequencing analysis. This could occur if the decrease in NCOs is not an actual decrease in the number of NCOs, but a decrease in the ability to detect NCOs given their shorter length. To address this issue, we calculated how many NCOs would be missed due to detection issues (Materials and Methods). For LO, 6.3+/−0.2% are missed. Thus as Spo11 activity declines, the ability to assess the true number of NCOs becomes more difficult and could influence calculations such as the NCO/CO ratio, though marginally.

Besides detection, alterations in tract length could have unexpected consequences on measurements of DNA repair. Tract length changes of NCOs and COs in opposite directions, might affect measurements of prototroph formation or the association of a reciprocal recombination with gene conversion.

### Modeling the tract length distributions in conjunction with CO homeostasis on *ARG4* DSB hotspots recapitulates genetic data

**Figure 5:**
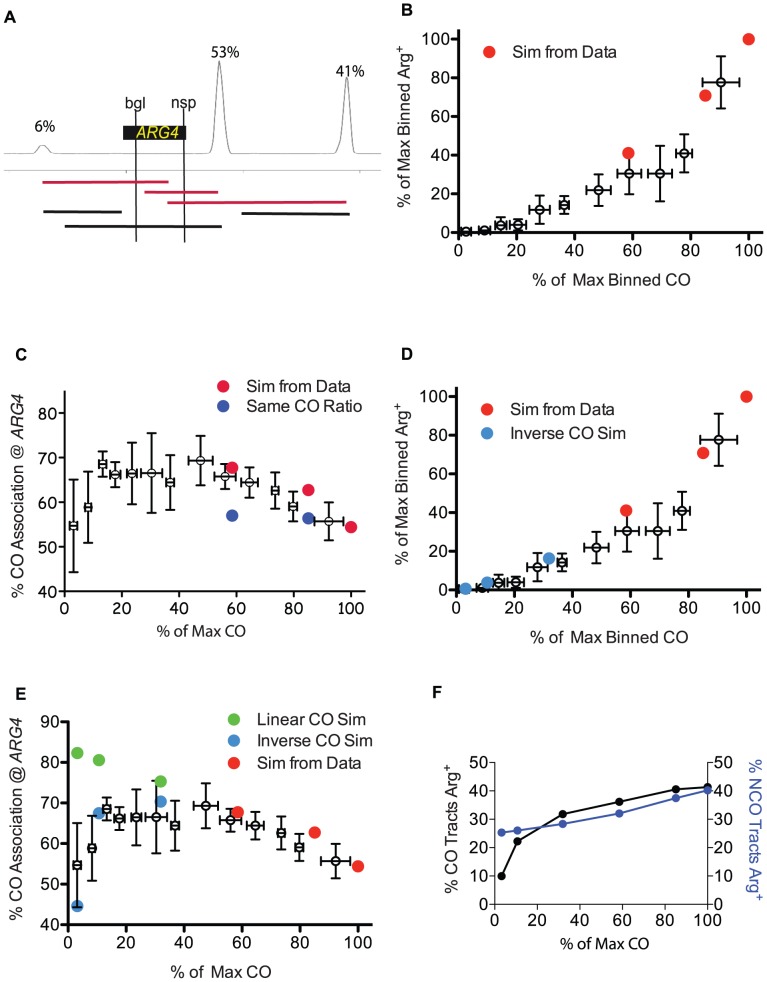
Simulation of genetic data from sequencing analysis. A. Diagram of the DSB location and usage around *ARG4*, showing three prominent hotspots with relative intensities of 6, 53 and 41 (left to right) [38]. The locations of the *ARG4* heteroalleles are shown; tracts that end between the alleles are capable of forming a prototroph. Examples of tracts that begin at a hotspot and end within the two heteroalleles are capable of forming an Arg+ are shown as red lines. Black lines depict examples of tracts that cannot form Arg+. B. Arg prototroph frequencies are plotted against normalized CO frequencies. Binned genetic data from Figure 3A is presented as empty symbols. Simulated values based on data from WT, HI and LO tetrads are indicated in red circles. C. The percentage of Arg prototrophs with a CO is plotted against CO frequencies. Binned genetic data from Figure 3C (empty symbols) are plotted with simulated data for WT, HI and LO tetrads (red circles). Dark blue circles represent simulations of the WT, HI and LO tract lengths but using the wild-type proportion of CO and NCO tracts. D. Same as B, with the addition of light blue circles representing mutants with 30, 10 or 3 COs as the result of extrapolating the tract length parameters (from WT, HI and LO) and performing the same simulations using an inverse regression for extrapolating the mean of the CO tract length distribution (see Materials and Methods). E. Genetic CO association data as in C, but including simulated values for virtual mutants with 30, 10 or 3 COs using an inverse regression (light blue circles) or linear relationship (green circles) (see Materials and Methods for calculations and explanation). F. Simulated CO and NCO tracts from mutants of varied Spo11 activity (WT, HI, LO, 30, 10 and 3) were scored for their ability to produce “virtual prototrophy” at *ARG4*.

In order to explore whether tract length changes in the *spo11* hypomorphs could account for the discrepancy between prototroph levels and crossing over, we tested if we could obtain the observed *ARG4* prototroph frequencies (Figure 3A) by computationally distributing tract lengths around the *ARG4* hotspot for each of the three levels of Spo11 activity. For this analysis, we took advantage of the detailed information available regarding the location and usage of meiotic DSB sites near *ARG4* [38]. Although these data were generated in the SK1 strain, we reasoned that the distribution of DSBs describes most genetic backgrounds since available comparisons with a hybrid strain used for microarrays and sequencing (YJM789xS96) have so far been in general agreement [7], [17], [19]. Information regarding tract length distribution and the relative abundance of NCOs and COs was used to generate sets of tracts for WT, HI and LO tetrads. Relative DSB frequencies at three hotspots in the vicinity of *ARG4* were taken from a DSB hotspot map derived from Spo11-oligos and were used as a template to position these tracts [38] (Figure 5A). We assumed that a tract initiating at one of the three hotspots and ending within the region between the two *ARG4* heteroalleles could generate a prototroph. We found that as Spo11 activity declined, projected prototrophs declined at an increased rate, comparable to the genetic results (Figure 5B, red dots).

We next asked if our tract length distributions could recapitulate the change in the fraction of Arg prototrophs associated with a CO that we observed genetically (Figure 3C). By determining which of the prototroph-forming tracts were associated with a CO and which were due to NCOs (Materials and Methods) a trend similar to that seen for the genetic results emerged, with increasing contributions from the COs as Spo11 activity declined (Figure 5C, red dots). The percentage of prototrophs associated with a CO increased from 54% in WT, to 63% in the HI tetrads, and 68% in LO tetrads (Figure 5C).

In Figure 5F, the proportion of NCOs and COs contributing to prototrophy as Spo11 activity decreases was obtained from the sequencing data (Figure S3A). Figure S3A indicates that as Spo11 activity decreases, COs represent a greater proportion of recombination repair, an observation consistent with the presence of CO homeostasis. However, is CO homeostasis necessary to attain the good fit to the genetic CO association data observed in Figure 5C? To address this question, we simulated tract lengths for our mutants (HI and LO) and generated values for CO association assuming no CO homeostasis, that is, we used the wild-type proportion of COs even when Spo11 activity declined. In the case of no CO homeostasis, the calculated values for CO association (Figure 5C, blue circles), did not match the genetic data, unlike the good fit achieved when CO homeostasis is incorporated (Figure 5C, red circles). We also looked at the effects of varying other parameters independently Figure S2.

### Modeling predicts severe GC tract length changes at the lowest levels of Spo11 activity

Can our experimentally generated genetic data be used to understand the relationship between tract lengths of NCOs and COs as Spo11 activity decreases to extremely low levels? The HI and LO tetrads examined above represent a limited range of Spo11 activity. It was not possible to obtain the sequence data needed to measure tract lengths for severe mutants because they do not produce tetrads with four viable spores. To circumvent this biological limitation, we performed simulations with various models of how tract length would change, extrapolating from the data of WT, HI and LO tetrads (Figure S3B–F). We then determined which of our simulations best describes our genetic observations.

First, we computed the number of NCOs as a fraction of total events in hypothetical mutants having 30, 10 and 3 COs, assuming a linear trend of the CO fraction as seen in our experimentally determined data (Material and Methods, Figure S3A). Second, we determined the mean tract length of log-normal CO and NCO distributions for these hypothetical mutants applying four different regression models of tract length behavior (Figure S3B–F, Materials and Methods).

We asked whether these models would recapitulate prototroph levels as a function of CO levels. All models tested were consistent with the aforementioned trend that, as COs declined, the frequency of prototrophs decreased at an increased rate, to as much as 7-fold lower than the decrease in COs (Figure 5D, data not shown).

We then asked which model would best recapitulate the genetic data with regard to the fraction of Arg prototrophs associated with a CO. Of all four regression models, only the inverse model for the mean CO tract distributions that predicts an inverse relationship between tract lengths and COs recapitulated the genetic data well (70%, 67% and 45% for simulated vs. 68%, 70% and 59% for genetic, for mutants undergoing 30, 10 and 3 COs, respectively) (Figure 5E). Moreover, although all models gave reasonable fits for Arg prototrophy frequency, the inverse model for mean CO tract distributions was the overall best (16%, 4% and 0.6% values vs. 12%, 1% and 0.5% genetic values, for mutants undergoing 30, 10 and 3 COs, respectively) (Figure 5D). Figure S3G shows that the inverse model for the CO tract length distribution predicts a severe increase in CO associated tract lengths in the cases where Spo11 activity is greatly diminished. This large increase in tract length in our model could explain why prototroph frequencies decline faster than CO frequencies in the measured genetic data as Spo11 activity declined. This suggests that as Spo11 activity decreases, gene conversion tracts associated with COs become too long to produce prototrophs, and NCO tracts become too short to produce prototrophs.

## Discussion

Analysis of nineteen new *spo11* hypomorphic alleles, encompassing a wide range of activity, revealed an unanticipated relationship between reciprocal recombination and gene conversion. Although the various *spo11* mutants displayed crossing over, SC and spore viability phenotypes that correlated well together, prototroph frequencies were far more influenced by the severity of the allele. This was an unexpected observation, since prototroph frequencies have commonly been used as a metric for DSB levels (e.g. [28], [39], [40]). High-throughput sequencing analysis of a subset of these mutants [6] showed that the fraction of COs (over total events) increased from 0.66 in WT to 0.77 in the LO tetrads (the most severe mutants), providing strong support for the existence of CO homeostasis.

Analysis of five tetrads from *spo11*-hypomorphs revealed extensive tract length alterations. Tracts associated with COs were longer in cells with diminished Spo11 activity. In contrast, NCO tracts were shorter in cells with lower Spo11 activity. By simulating tracts changing both in length and in the ratios of CO and NCOs for the mutants and then placing them onto the well-characterized *ARG4* DSB map [38], we recapitulated the result that prototroph levels are more affected than CO levels in severe *spo11* alleles. Modeling the altered tracts onto the *ARG4* locus clarifies both the upward trend of increasing CO association for the less severe *spo11* alleles and the downward trend in CO association that occurred with more severe *spo11* alleles. Both the changes in tract length and CO/NCO ratio are needed to generate the best fit to the genetic data (Figure S2).

Analysis of hypomorphic mutants of the *SPO11* homolog in *S. pombe*, *REC12*, revealed similar unexpected relationships between the severity of certain hypomorphs and recombination outcome [41], complementary to what we report here. Intragenic recombination was reduced even in mutants that appeared not to have a significant reduction in DSBs or COs. The authors conclude that these alleles reveal a “separation-of-function”. However, in light of the present study, we propose an alternative explanation, that *S. pombe REC12* mutants experience changes in tract lengths that would impact measures of intragenic recombination similar to what we find at *ARG4*.

### Models for tract length changes

Tract lengths associated with COs become longer as Spo11 activity declines. Longer tracts may be due to an increase in branch migration and resection [42]. Crossover recombination is thought to occur at SICs, which, like recombination nodules, contain many of the enzymes thought to be necessary for catalyzing the CO [43], [44]. In *spo11* hypomorphs, there are fewer SICs, as estimated by numbers of Zip3 foci [14]. We propose that, in the *SPO11* hypomorphs, recombination enzymes, including those for resection and mismatch repair, are distributed between fewer SICs, thus enriching them with a relative abundance of enzymes. Consequently, these SICs may be more active, and generate longer resections. This proposed increase in enzymatic ability in hypomorphs with low Spo11 activity might increase in a nonlinear way, reflecting the predicted increases in tract lengths. A similar explanation was given to explain the long resection products of a single VDE cut in a *spo11* null background [45], [46]. Efficient DSB formation at a site in an otherwise DSB-deficient strain (*spo11*-null) produced longer resected tracts and generated fewer prototrophs than expected, and this phenomenon was apparently due to reduced levels of DSBs in the cell (and not synapsis defects) [45]. Similarly, Malkova et al. [47], [48] saw increased GC tracts from HO-induced DSBs in strain backgrounds with no meiotic DSBs.

In contrast to GC tracts associated with COs, NCO tract lengths are shorter in cells with diminished Spo11 activity. Most NCOs are thought to occur by a different mechanism than COs which may help to explain this altered outcome. While COs go through a double Holliday junction intermediate, NCOs are thought, for the most part, to undergo a single strand invasion or SDSA (Synthesis-Dependent Strand Annealing) [16]. SDSA events are likely independent of SICs, since the number of these events is not reduced in mutants lacking SIC components [49], [50]. We propose that the reduction in NCO tract length is due to SDSAs forming in the absence of stably associated homologs. That is, the Spo11 hypomorphs have reduced levels of homolog pairing and SC ([14], this paper), which may otherwise stabilize the interaction of non-sister chromatids. We propose that SDSA recombination is sensitive to this lack of reinforced homolog alignment. In the absence of stable associations, the movement of chromosomes in the prophase nucleus [51] could interrupt the SDSA event prematurely, leading to even shorter gene conversion tracts. Nuclei with less Spo11 activity and fewer paired chromosomes would have the greatest obstruction to SDSA events.

### Evidence for CO homeostasis

Our data raise important considerations regarding the characterization of CO homeostasis.

First, sequencing of *spo11* hypomorphic tetrads revealed an overall reduction in recombination events, with a stronger reduction in NCOs, supporting the notion of CO homeostasis. However, since NCO tracts in strains with limiting DSBs are shorter, these data may reflect an inability to detect NCOs due to their lesser likelihood to span a heterologous SNP. We have estimated the loss of NCOs in the mutants by positioning tracts on the genome taking into account the actual SNP density map. In the case of our most severe hypothetical mutant (3CO) up to 16% of the NCOs become undetectable yet this apparent loss of NCOs only marginally affects measures of CO homeostasis. An alternative explanation for the reduction of NCOs, particularly in severe mutants where homolog pairing is severely compromised, may be that some DSBs fail to find their homologous partner and are instead repaired using the sister chromatid [52].

Second, the assay used to measure CO homeostasis, flanking marker exchange of prototrophs, assumes that a tract emanating from a CO or an NCO would have the same probability of forming a prototroph. However, since tract lengths change in unexpected ways in both NCOs and COs, and thus alter their abilities to form prototrophs, apparent flanking marker exchange cannot be assumed to be equal, particularly in severe hypomorphs. Note that different pairs of heteroalleles and different loci will elicit unique outputs.

Using a novel color system to study CO homeostasis in tetrads, Thacker et al. [18] were unable to detect CO homeostasis in a flanking marker assay in the *spo11-*HA mutant which has little impact on DSB levels. The authors argued that tract-length alterations might obscure NCO/CO ratios. Consistent with this, if we apply our observed tract length changes to the GFP alleles, we would expect a decrease of GFP^+^ recombinants associated with COs and an increase in GFP^+^ recombinants unassociated with a CO, since the heteroalleles are so close in this assay. So although our measurements of tract length in the HI mutants may not be significant, it seems reasonable to suggest that tract lengths are changed in modestly defective *spo11* alleles in both budding and fission yeast (see above).

Consistent with the operation of CO homeostasis, we were only able to recapitulate the CO/NCO genetic data when applying the experimentally observed changes in numbers of CO and NCOs. Thus our data support the accumulating evidence that as Spo11 activity decreases, the fraction of recombination events that become COs increases.

### Rescue by Ndt80 arrest

Our observation that two severe *spo11* alleles can be rescued for SC formation, recombination and spore viability by the *ndt80* mutant suggests that holding cells in prophase allows further DSB formation. This notion, that DSB formation may continue in cells held at mid prophase, has been suggested previously [53], based on limited increases in prototroph formation of arrested cells. Further support came from the observations of Allers and Lichten [16] in which they report an accumulation of joint molecules during Ndt80 arrest. However, we find that WT diploids arrested at the Ndt80 arrest point fail to show genetic evidence of the additional DSBs in spores (i.e., no increase in heteroallelic recombination or crossing over, Figure 2).

If continued DSB activity is the result of Ndt80 arrest, then why is the genetic consequence strongly observed in certain *spo11* hypomorphs, but only minimally in WT? Two scenarios seem likely. In the first, DSBs are unregulated (occurring in all backgrounds), but the repair outcome is dependent on the state of synapsis. In synapsed chromatin, the repair would favor the sister (SC is inhibitory to interhomolog recombination) and in unsynapsed chromatin, interhomolog repair would be favored. For poorly synapsed mutants, Ndt80 arrest would result in a disproportionate increase in observable recombination (and consequently increased synapsis). This model, where stably paired and/or synapsed chromatin is protected in *cis* from additional interhomolog repair, has been previously suggested to account for observations in worms [54]. A second possibility has been suggested for mouse meiosis, where DSB formation may be limited within synapsed chromatin [55]. If DSB activity, rather than repair, is modulated, such that unsynapsed chromatin receives more DSBs than already synapsed chromatin, and the DSBs are repaired normally (in favor of inter-homolog repair), this would also disproportionately affect meioses with poor synapsis. In either case, how DSBs and their repair may be controlled gives insight into how the meiotic cell may ensure higher levels of pairing, SC formation and recombination in meiotically compromised cells.

## Materials and Methods

### Screen for non-null mutants

Mutants of *SPO11* were generated by a degenerate PCR based method. First, the endogenous *SPO11* gene from our strain background (BR1919-8B) was cloned and sequenced, and it was found to encode six altered amino acid changes compared to that reported for S288C (SGD), although it had the identical amino acid sequence to SK1, another strain often used to study meiosis. The sequence of the *SPO11* gene contains a string of 12 As which can corrupt the integrity of its sequence during PCR amplification. To alleviate this problem, two As in the middle of the run were silently substituted with Gs (A225G, A228G). A centromere plasmid containing this functionally wild-type *SPO11* gene and *URA3* (pRS316:*SPO11*) was subjected to degenerate PCR amplification (GeneMorphII EZClone Domain Mutagenesis kit from Stratagene). Three independently mutagenized plasmid libraries were prepared and transformed into a *spo11-null* diploid yeast strain (BR4590) for screening (Table S6).

In order to identify non-null alleles of *SPO11*, assays for both prototrophs at the *LEU2* and *THR1* loci, and spore viability (Can^R^ Cyh^R^) were incorporated into the diploid yeast strain used for the screen (see Table S6). Using this strain, null mutants, after sporulation would form no or very few papillae on either the leucine or threonine omission medium or the drug-containing medium after sporulation. Both cold-sensitive and heat sensitive alleles were isolated by screening replicas sporulated at different temperatures (22° and 30°C).

Over 26,000 transformants were screened from the three independent mutagenized libraries. 10.8% of the screened transformants behaved as null mutants, indicating that the mutagenesis was successful. Those passing a second screening (52 mutants) were chosen for plasmid retrieval, sequencing and retesting by retransformation into BR4590. The mutations were distributed throughout the gene (Figure S1). Six mutants were isolated more than once and at least five of these were independent. Nine of the mutants encoded a single amino acid change, but we chose to include eight alleles with two amino acid changes and two with three changes in our analysis.

### Genetic analysis

The sequenced alleles were subcloned into integrating vectors (pRS306) so that they could be transformed stably into yeast by pop in/pop out and studied as homozygotes. Crosses to generate the various strains utilized a marked *SPO11::KAN (*or *SPO11::HYG* or *SPO11::NAT)* allele to monitor the unmarked mutants. All strains for subsequent analysis are isogenic to BR1919-8B [3] and any markers were changed by transformation. Isogenic derivatives were used for the subsequent analysis (Figure 1A, BR5348 and it's *spo11-m* derivatives Table S1, S6).

For nineteen of the new mutants and one previously described (D290A [28]), arginine prototroph frequencies and map distances for three intervals on chromosome 3 were obtained from cultures sporulated at 18 and 22, and 31.5°C. Prototroph frequencies were averaged from at least three cultures and premeiotic frequencies were determined and subtracted. Map distance measurements in dyad spores are complicated by occasional reductional segregation and the fact that recessive phenotypes can be masked by the dominant allele. Map distances for chromosome 3 markers were determined by first sorting out all the phenotypic classes. Although several classes of events are hidden by heterozygosities in one spore, their sister spore would be scorable. Equal numbers of scorable genotypes were then subtracted from the hidden classes and added to the appropriate recombinant class. Then CO's are summed for each interval to provide map distances.

In a subset of mutants (*spo11-32*, *179* and *217*) and WT, *LEU2* heteroalleles were added to the strains to measure prototrophs at a second locus. The *leu2-3,112* allele already exists in the strain background. To create a second allele, a haploid was first transformed to Leu^+^ and subsequently transformed to *leu2-cla* by pop-in pop-out transformation (R477: pBR322 with *Eco*RI/*Xho*I fragment of *LEU2* where the *Cla*I site of *LEU2* was filled in with Klenow). *HIS4* and *iHYG* flank the *LEU2* gene.

Strains for the sequence analysis were heterozygous for the *spo11* alleles. The YJM789 strain is deleted for *spo11* and constructed by transformation of *ura3::NAT* derivatives of the two haploids, S96 and YJM789 [37], with pRS306-*spo11-32* and pRS306-*spo11-217* (and subsequent popout). Zygotes were isolated and sporulated at various temperatures. Tetrads were used from the *spo11-217* strain from dissections at 31.5°C (spore viability was 81%; 78/96) and 24°C (spore viability was 11%; 65/576) and from the *spo11-32* diploid, tetrads were used from dissections at 30°C (spore viability 10%; 58/576 with only four 4-spore viable tetrads). Wild-type hybrid strains were examined for temperature effects by sporulation at 22°C, 26°C and 30°C. Wild-type sequences are stored at the National Center for Biotechnology Information Sequence Read Archive (Bioproject accession number SRP028549). Output files from Recombine, CrossOver and TractSeq are available from the Dryad Digital Depository: http://doi.org/10.5061/dryad.53t4c.

### CO association analysis

The fraction of Arg prototrophs resulting from a CO was determined from analysis of random dyad spores. Flanking markers, *THR1* (4 cM distal to *arg4-Nsp*) and *iTRP1* (a marker placed near the centromere, 16 cM proximal to *arg4-Bgl*) were scored for nonparental segregation among Arg prototrophs. Since the most frequently converted allele is cis to the Trp^+^ marker, most of the Arg^+^ spores are Trp^+^ and the reciprocal recombinants are Thr^−^. The number of Trp^+^ Thr^−^ recombinants were doubled to account for the inability to see the reciprocal event.

A similar assay for CO association was carried out on chromosome 3, utilizing *LEU2* heteroalleles and using the hygromycin resistance marker at *CEN3* and the distal marker, *HIS4* as flanking markers (Figure 1A). The configuration of the *LEU2* alleles and the flanking markers is best suited for detecting recombinants (*leu2-cla* converts ∼80% of the time and thus is flanked by both HygR and *HIS4*) Table S4.

### CHEF gels

Plugs were prepared from meiotic cultures and subjected to CHEF gel analysis [29] and Southern hybridization with a probe from chromosome 3 sequences (*THR4* region) prepared from random priming kit (Rediprime II, GE Healthcare) and analyzed on a Storm phosphorimager (GE Healthcare). The “fraction recombinant bands” was estimated by summing twice the intensity of the trimer band plus the dimer band over the total intensity of the three bands. The averages of five experiments are presented. For the DSB analysis, plugs were made, ran, and hybridized as above, but from *sae2Δ* versions of the mutants. Three gels were averaged.

### Meiotic chromosome spreads

Meiotic chromosomes were spread according to Rockmill [56]. Cultures sporulating at 31.5°C were spread after 15 hours, those sporulating at 22°C were spread at 24 hours and those sporulating at 18°C were spread at 30 hours to maximize those cells with the most SC. Since sporulation in this strain background is not synchronous, Red1 staining was used to identify nuclei at a similar stage, mid prophase I. Red1 accumulates on the chromosomes from early in prophase and culminates at the stages where the SC formation is most abundant, and is lost by Meiosis I. SC is scored by visualizing the Zip1 protein by immunofluorescence. *spo11* mutants often form an aggregate of Zip1 and other proteins called a polycomplex (PC). In our strain background WT does not normally form PCs. SC was scored among Red1 positive nuclei as follows: One or no Zip1 lines (the PC can appear linear) is “no SC”; Two or more Zip1 lines but fewer than complete SC is “some SC”; and Zip1 lines encompassing the entire nuclear region is “full SC”. Raw data is presented in Table S2.

### Multiplex tetrad sequencing

DNA from four-spore viable tetrads was purified and processed for Illumina high-throughput sequencing. Illumina sequencing libraries were generated using adapters for multiplexing, as described [57]. In general, four barcoded libraries, one from each spore of an individual tetrad, were mixed in equimolar ratios and processed on an Illumina Genome Analyzer II. One tetrad was analyzed using a two-plex strategy. Each sequence read started with a 4-bp index followed by 32 bp from the sample. Raw sequencing data were first processed by Illumina's Casava pipeline. After barcode parsing, the remaining bases were aligned against the S96 and YJM789 genomes using ReadAligner [6]. Barcode sorting, alignment to reference genomes, genotyping and detection of recombination events were performed using a suite of programs included in ReCombine [6].

### Determination of tract lengths from sequencing data

A pillar of our analysis rests on a stringent and accurate estimation of gene conversion tracts for CO's and NCO's among various tetrads. Again the ReCombine package was employed, with a few modifications. First, we examined marker calls consisting of SNPs and indels to ensure the use of high-quality heterologies and to remove markers that might be misannotated and thus assigned to the wrong parent genome. A “confusion matrix” was created by comparing quality scores against S96 and YJM789 genomes from sequenced S96 and YJM789 parental strains, an important metric during genotype calling. For example, from a sequenced S96 parent, if a SNP had a higher quality score for YJM789 than S96 or equal scores, this heterology would be classified incorrectly as an YJM SNP. This ambiguity would lead to false positives and incorrectly called events and tracts. The estimated error rate was 0.3% for SNPs and 29% for indels after a first-pass analysis; these markers were excluded. Nevertheless, some single marker events encompassed an indel with mixed reads that passed our first-pass thresholds. Sanger sequencing of genomic DNA from spores failed to validate 13 out of 14 such events. We limited our analysis to SNP marker calls given the higher genotyping errors found in indels.

Second, marker calls were made on a tetrad basis and on individual spores to increase the precision of tract length predictions. The ReCombine package determines events and tracts considering all four spores [6]. It excludes from analysis high-confidence heterologies that could not be called in any one of four spores in a tetrad, often due to reduced local sequencing depth (no sequence read spanning the SNP). Using as a template the recombination map generated by ReCombine, we confirmed these events in a separate single spore analysis. This approach better defined tract lengths, by extending tract lengths, adding better boundaries to existing tracts and increasing the number of heterologies defining a given event. Moreover, our strategy uncovered a handful of recombination events that were missed by tetrad-based analysis. Overall, tetrad-based and individual spore analyses gave very similar results.

Tract length boundaries were predicted using the individual spore procedure described above. The minimal tract length was defined by markers that are certain to be part of the gene conversion event, while the maximal tract length was defined by the next 2∶2 segregating flanking markers, before which the gene conversion tract must end. The difference between the maximal and minimal tract lengths is often large, leaving the actual tract length ambiguous. Rather than using a commonly used measure, such as the midpoint, we have developed an algorithm to better estimate the tract lengths, taking into account biologically relevant information such as enzymatic processivity and marker distribution asymmetry (Figure 4A). Processive enzymes have a probability “p” of moving to the next base, and a probability “1-p” of falling off, following a geometric distribution (or exponential law). Statistical approaches have been able to model gene conversion tracts from genetic data using a maximum likelihood approach, considering ressection as a succession of Bernoulli trials [58]. Assuming bidirectionality from an initiation site, tract lengths would then follow a log-normal distribution [58], [59]. In Figure S4 we show the results using unidirectional compared to bidirectional tract formation. From gene conversion data obtained by genetic means, enzymatic processivity parameter “p” has been estimated to be around 0.999 in flies, yeasts and mice, from a meta-analysis [59]. Sequencing data differ from genetic assays in the measurements of tract lengths. In genetic analyses, an initiation site, i.e. the position of a DSB hotspot, is often known, and gene conversion gradients are usually measured from one side, establishing tract lengths that may only reveal half the length. Whereas a conversion tract determined by sequencing consists of a central region of certainty (minimal tract length) flanked by regions of uncertainty (maximal tract length). We developed a novel analytical method, Tract-Seq, to estimate more accurately the lengths of gene conversion tract, or any marker-based estimation of enzymatic processivity from sequencing data, using Monte Carlo simulations (Figure 4A). For each iteration, the bidirectional enzymatic complex falls from the end of the minimal interval, using an exponential law decreasing away from the minimal tract on both sides of the interval. These are termed endpoints. Tracts extending past the maximal gene conversion interval were not considered as were those not extending past the minimal gene conversion interval. We assumed a similar probability “p” as the enzymatic processivity (0.999) [59]. Using p values of 0.9970 to 0.9993, showed indistinguishable trends in tract lengths, suggesting these trends are robust to slight perturbations of enzyme processivity. The estimated tract length was then represented by the sum of the following distances: endpoint 1 to minimal tract (start) + minimal tract + minimal tract (end) to endpoint 2. After a minimum of 10,000 iterations, the median length of the estimated tract was used for distribution fitting. Statistical analysis of the median of tracts was performed using a non-parametric resampling test.

For events that span a single marker, a minimal interval cannot be determined with this method. This is often the case in regions with low marker density. As an example, a maximal interval might be 20 kb while a minimal interval of 1 bp. In such cases, using the maximum likelihood estimation from geometric distribution with processivity “p” described above to fine-tune estimated tract lengths, intervals of similar length would be generated. Since these point events are common (∼15%), it would result in biases during distribution fitting. To circumvent this problem, we made an estimation of tract length for tracts defined by a single SNP. From the linear relationship between maximal tract lengths and tract lengths defined by multiple SNPs, we determine an approximation for the tract length for a single SNP tract based on its maximal tract length.

### Distribution fitting

To ensure a better overall fit, tract lengths from COs and single tract NCOs were grouped by their levels of Spo11 activity: WT, HI and LO. Tracts from complex events, such as NCOs on two chromatids and NCOs associated with a CO, display longer tract lengths than regular COs and NCOs, and these types were treated separately (and *SPO11* genotypes were pooled). Gene conversion tract lengths can be described by a log-normal distribution [58], [59] (see Text S1). Log-normal curves were fitted by a Bayesian analysis with Markov Chain Monte Carlo (MCMC), using the R package – rjags (JAGS, http://mcmc-jags.sourceforge.net/) to determine the mean and standard deviation of log normal distributions. We tested the goodness of fit using Kolmogorov-Smirnov tests and could not reject any fitted curves from tract length data points (P>0.05 in all cases). Significance in the differences of the means was tested using re-sampling by comparing actual means along with the corresponding error on the means obtained from MCMC iterations (sd_mean_), correcting these sd_mean_ for sample size differences (i.e. different numbers of tract lengths (data points) used in the distribution fitting process).

### Estimation of NCO loss due to inability to detect shorter conversion tracts

To estimate the percentage of NCOs that would be missed because of shorter conversions tracts, we distributed onto our SNP map, a wild-type number of NCO tracts that were sampled from the fitted tract length distributions for WT, HI and LO. The percentage of NCO tracts that span at least one SNP was recorded. The difference between the percentage detected in WT vs. HI and WT vs. LO estimates the change in NCO numbers due to detection issues.

### Computational analysis of *ARG4* prototroph formation from gene conversion tract distributions

Gene conversion tracts were generated from fitted CO and NCO log normal distributions for WT, HI and LO. Similarly, tracts were created for severely hypomorphic *SPO11* mutants from log normal curves using inferred parameters, as described above. We assume that genome-wide averages hold for any individual site. Tracts originated from one of three DSB sites around the *ARG4* locus, in proportions that parallel the frequency of DSBs found by Spo11 oligo sequencing [38]. A tract originating from one initiation site and of length falling between the Bgl and Nsp heteroalleles is classified as prototroph-forming (Figure 4A). DSB site 1, located downstream of *ARG4*, is the initiation site for 6% of simulated tracts, which form prototroph if they are 1544 to 2818 bp in length. DSB sites 2 and 3, upstream of *ARG4*, accounts for 53% and 41% of initiations [38] and tracts emanating from these sites can generate a prototroph if they are comprised between 204 and 1478 bp for DSB site 2, or between 2354 and 3628 bp for DSB site 3. Monte Carlo simulations were performed to record the average number of prototroph-forming events and its standard deviation for different classes of events emanating from one of three DSB sites. For each of 1,000 iterations, more than a million random tracts were generated from various CO and NCO log normal distributions, totaling more than a billion tracts considered per genotype. The number of prototroph-forming tracts was then adjusted to follow the number of various types of meiotic recombination events for *SPO11* hypomorphs. The percentage of prototroph-forming tracts from NCOs and prototroph-forming tracts originating from a CO event were calculated. To determine the individual effect of the parameters used, we varied each parameter separately: biased usage of *ARG4* hotspots, NCO/CO ratio and tract length variations (Figure S2).

### Extrapolation of tract length distributions and meiotic recombination events for severely hypomorphic *SPO11* mutants

Hypothetical mutants with 30, 10 and 3 COs were designated as highly hypomorphic, (for which four-spore viable tetrads could not be experimentally obtained). Number of COs with tracts was estimated at 71% from pooled WT, HI and LO tetrads. Assuming a linear decrease in the proportion of NCOs as Spo11 activity diminishes (CO homeostasis), the number of total NCO's was estimated at 5.5, 1.2 and 0.3 for mutants with 30, 10 and 3 CO's respectively, using a linear regression for the fraction of CO's in total events from tetrad sequencing data. To determine the fraction of “complex events” (i.e., NCOs that appear as two COs and GC associated with a CO on a chromatid not involving the CO), the average proportion across WT, HI and LO was used. Complex NCO events are 14% of total NCO events and each event consists of two distinct tracts. Similarly, 7% of total CO's include a conversion tract on a third chromatid. In analyses involving the absence of homeostasis, the number of NCO events was kept constant at the same fraction of total events as in WT.

Means and standard deviations of log normal CO and NCO distributions for simulated mutants were first inferred using linear models lm(mean ∼ #CO) and lm(sd ∼ #CO). Given that the CO association at *ARG4* from *in silico* analysis did not follow the trend seen in genetic assays due to the overproduction of prototrophs from CO tracts, we performed non-linear regressions to increase the means of CO curves and obtained overall better fit compared to a simple linear regression. Specifically, the following non-linear models were used during extrapolation: lm(mean ∼ √#CO), lm(mean ∼ log(#CO)) and lm(mean ∼ 1/#CO). The latter one provided the best fit to genetic data for CO association and prototroph formation frequency at *ARG4*.

## Supporting Information

Figure S1 Description of *spo11* alleles. A) The alignment of *SPO11* to *Methanococcus jannaschii* topoisomerase VI subunit A (Nichols et al., 1999) was used to locate the *spo11* alleles into known features of the topoisomerase VI subunit A crystal structure. Molecular graphics and analyses were performed with the UCSF Chimera package (http://www.cgl.ucsf.edu/chimera). The model (TopVI_map100_labels.py) is found at Dryad Digital Depository: http://dx.doi.org/10.5061/dryad.[NNNN]. Subunit A is represented as the tan ribbon structure with the 5Y-CAP domain highlighted in green while the Toprim domain is highlighted in yellow. The tyrosine active site is colored magenta within the 5Y-CAP domain. Blue residues mark cold-sensitive *spo11* alleles. Red residues are heat-sensitive alleles. Orange alleles fluctuate between cold and heat sensitivity depending on the temperature. The white allele is insensitive to temperature. The Mg2+ metal ion in the Toprim region is shown as a small green sphere. Subunit B is colored in gray. B) *spo11* alleles are listed alongside their affected residues, their temperature sensitivities, whether the residues are conserved and their location in the protein domain. Symbols show which residues correspond with which domain. “Varies” means that the residue can be cold and heat sensitive depending on the temperature range. No structural alignment means that the crystal structure was unavailable. Conservation refers to the original residue.
(EPS)

Figure S2 Contribution of CO and NCO ratios and tract length changes on simulations. Black lines indicate values obtained for simulations using the predicted (and experimental) changes in CO/NCO ratio as well as tract length changes for simulated prototroph frequencies (A and C) and CO association (B and D). Prototroph frequencies (A) and CO association frequencies (B) were simulated using the three *ARG4* region hotspots equally for initiation (red lines). Prototroph (C) and CO association frequencies (D) were simulated using wild-type tract length parameters either using predicted changes in CO/NCO ratios (red lines) or constant ratio (blue lines).
(EPS)

Figure S3 Extrapolated data for mutants with 30, 10 or 3 COs. A, COs as a percentage of total events were plotted against COs per tetrad based on sequence data from WT (black), HI (red) and LO (blue) tetrads. Four-chromatid double COs were counted as single events; in addition, both CO and NCO events in which there was a GC tract on two chromatids were counted as single events. A line (linear regression) was drawn through the points (R^2^ = 0.6), indicating the trend of more COs per total events as Spo11 activity declines. B–F, Distributions of GC tract lengths associated with NCOs (B) and COs (C–F). Log-normal curves were fitted for WT, HI and LO to determine the underlying distribution parameters (means and SDs). Mean tract lengths were then extrapolated for strains with 30, 10 or 3 COs, using either a linear regression (B and C) or various non-linear regressions including square-root (D), log (E) or inverse (F) functions of X (X being the number of COs). In all cases, SDs of GC tract length distributions were obtained using linear trends. CO tract length log-normal distributions for WT (solid black line), HI (red), LO (blue), and 30 COs (green), 10 COs (purple) and 3 COs (yellow) simulated mutants are plotted, along with their respective means (dotted lines). G. Representation of linear and non-linear regression curves (linear, black; square-root, red; log, blue; inverse, green) used to extrapolate the mean of the CO tract length distributions for putative mutants with 30, 10 and 3 COs (dotted vertical lines). The x-axis represents the number of COs while the y-axis represents the mean of CO tract length distributions in base pairs.
(EPS)

Figure S4 Half vs. whole tracts. Black lines indicate values obtained for simulating Arg prototrophs (A) and CO association (B) using tracts initiating at the DSB hotspots. Red lines similarly show values for “half tracts”, in which the DSB hotspot would fall in the middle of the tract.
(EPS)

Table S1 Arg prototroph data.
(XLSX)

Table S2 Spore viability and SC formation.
(XLSX)

Table S3 Crossing-over in strains released from Ndt80 arrest and WT.
(DOC)

Table S4 Leu prototroph data.
(XLSX)

Table S5 Sequencing data for wild-type tetrads at various temperatures.
(XLSX)

Table S6 Strain list.
(DOCX)

Text S1 Methods.
(PDF)
