# Correction: High Throughput Sequencing Reveals Alterations in the Recombination Signatures with Diminishing Spo11 Activity

**DOI:** 10.1371/annotation/b3e65d4a-f7ed-4481-9b5b-92d952d5ee02

**Published:** 2013-12-20

**Authors:** Beth Rockmill, Philippe Lefrançois, Karen Voelkel-Meiman, Ashwini Oke, G. Shirleen Roeder, Jennifer C. Fung

There was an error in the citation information for reference [41]. The correct citation is “Kan F, Davidson MK, Wahls WP (2011) Meiotic recombination protein Rec12: functional conservation, crossover homeostasis and early crossover/non-crossover decision. Nucleic Acids Res 39:1460-1472.”

In the discussion section, the sentences “The authors conclude that these alleles reveal a “separation-of-function”. However, in light of the present study, we propose an alternative explanation, that *S. pombe REC12* mutants experience changes in tract lengths that would impact measures of intragenic recombination similar to what we find at *ARG4*.” should be changed to:

"Our interpretation of the data in Kan et al. (2011) leads us to alternatively conclude, in light of the present study, that *S. pombe REC12* mutants experience changes in tract lengths that would impact measures of intragenic recombination similar to what we find at *ARG4*."

