# Supplementary material for: High Throughput Sequencing Reveals Alterations in the Recombination Signatures with Diminishing Spo11 Activity
Source: PLoS Genet. 2013 Oct 31;9(10):e1003932. doi: 10.1371/journal.pgen.1003932 (PMC3814317; doi:10.1371/journal.pgen.1003932)
Supplement: Table S3 — Crossing-over in strains released from Ndt80 arrest and WT. (DOC) [file pgen.1003932.s007.doc]

**Table S3**

| Strain | Genotype | *HIS4-CEN*  (PD-NPD-TT)  cM | *CEN-MAT*  (PD-NPD-TT)  cM | *MAT-iADE*  (PD-NPD-TT)  cM |
| --- | --- | --- | --- | --- |
| BR4901 | *SPO11 NDT80* | (160-1-170)  26.6 | (196-5-133)  24.4 | (121-5-202)  35.4 |
| BR5862 | *SPO11 NDT80-E* | (49-3-55)  33.5 | (57-2-47)  27.1 | (50-5-46)  34.9 |
| BR5864 | *spo11-217 NDT80-E* | (76-0-38)  16.7 | (55-5-54)  36.8 | (83-0-29)  12.9 |

Crossing-over in strains released from Ndt80 arrest and wild type. Diploids were incubated in sporulation medium at 18^o^C for 48 hours and then induced to sporulate by the addition of estrogen to induce *NDT80* expression. Tetrads were dissected and at least 100 four-spore viable tetrads were dissected and scored for recombination for three intervals on chromosome 3.
