# Supplementary material for: High Throughput Sequencing Reveals Alterations in the Recombination Signatures with Diminishing Spo11 Activity
Source: PLoS Genet. 2013 Oct 31;9(10):e1003932. doi: 10.1371/journal.pgen.1003932 (PMC3814317; doi:10.1371/journal.pgen.1003932)
Supplement: Table S6 — Strain list. (DOCX) [file pgen.1003932.s010.docx]

**Table S6. Strains used in this study**

| **NAME** | **GENOTYPE** |
| --- | --- |
| **BR4590** | *MATα leu2-3,112 his4-260,519 spo11Δ::ADE2 thr1-4 ade2-1 ura3-1 trp1-289*  *MAT****a*** *leu2-27 his4-290 spo11Δ::ADE2 thr1-1 ade2-1 ura3-1 trp1-1*  *CYH3 can1R*  *cyh3R CAN1* |
| **BR4901** | *His4-Hpa HYG@CEN3 MAT****a*** *ADE2@RAD18 spo13::URA3 arg4-Bgl thr1 lys2 trp1-289*  *HIS4 MATα SPO13 ARG4 THR1 LYS2 TRP1*  *ade2-1 ura3-1*  *ade2-1 ura3-1* |
| **BR5229** | *his4-260,519 leu2-3,112 HYG@CEN3 MATα sae2 ade2-1 ura3-1 trp1-289 thr1-4*  *Circle3 HIS4 leu2-3,112 CEN3 MAT****a*** *sae2 ade2-1 ura3-1 trp1-289 thr1-4* |
| **BR5230** | *his4-260,519 leu2-3,112 HYG@CEN3 MATα ade2-1 ura3-1 trp1-289 thr1-4*  *Circle3 HIS4 leu2-3,112 CEN3 MAT****a***  *ade2-1 ura3-1 trp1-289 thr1-4* |
| **BR5231** | Same as BR5229, but homozygous *spo11::ADE2* |
| **BR5232** | Same as BR5230, but homozygous *spo11::ADE2* |
| **BR5233** | Same as BR5229, but homozygous *spo11-217* |
| **BR5234** | Same as BR5230, but homozygous *spo11-217* |
| **BR5235** | Same as BR5229, but homozygous *spo11-179* |
| **BR5236** | Same as BR5230, but homozygous *spo11-179* |
| **BR5237** | Same as BR5229, but homozygous *spo11-32* |
| **BR5238** | Same as BR5230, but homozygous *spo11-32* |
| **BR5348** | *his4-260,519 leu2-3,112 HYG@CEN3 MATα ADE2@RAD18 ade2-1 ura3-1 trp1-289*  *HIS4 leu2-Cla1 CEN3 MAT****a*** *RAD18 ade2-1 ura3-1 trp1-289*  *TRP1@CEN8 spo13::URA3 arg4-Nsp THR1*  *CEN8 spo13::URA3 arg4-Bgl thr1-4* |
| **BR5340** | Same as BR5348, but homozygous *spo11-217* |
| **BR5341** | Same as BR5348, but homozygous *spo11-179* |
| **BR5349** | Same as BR5348, but homozygous *spo11-32* |
| **BR5408** | Same as BR5348, but homozygous *SPO11:KANMX@Swa1* |
| **BR5418** | Same as BR5348, but *SPO11*  *spo11::ADE2* |
| **BR5431** | Same as BR5348, but *SPO11:KANMX@Swa1*  *spo11::ADE2* |
| **BR5438** | Same as BR5348, but *SPO11:KANMX@stop codon*  *spo11::ADE2* |
| **BR5361** | *ndt80Δ::LEU2 MATα leu2-3,112 his4-260,519 ade2-1 ura3-1 trp1-289 thr1-4*  *ndt80Δ::LEU2 MAT****α*** *leu2-Cla his4-260,519 ade2-1 ura3-1 trp1-289 thr1-4* |
| **BR5362** | Same as BR5361, but homozygous *spo11-217* |
| **BR5363** | Same as BR5361, but homozygous *spo11-179* |
| **BR5364** | Same as BR5361, but homozygous *spo11-32* |
| **BR5862** | *his4-260,519 leu2-3,112 HYG@CEN3 MATα ADE2@RAD18 ade2-1 ura3-1 trp1-289*  *HIS4 leu2-3,112 CEN3 MAT****a*** *RAD18 ade2-1 ura3-1 trp1-289*  *_pGAL_-NDT80@TRP1 _pGAL4_ER@URA3 arg4-Nsp THR1*  *_pGAL_-NDT80@TRP1 _pGAL4_ER@URA3 arg4-Bgl thr1-4* |
| **BR5863** | Same as BR5862, but homozygous *spo11-179* |
| **BR5864** | Same as BR5862, but homozygous *spo11-217* |
| **S96** | S96 *MAT****a*** *ho lys5* |
| **S96-217** | Same as S96, but *spo11::HYG ura3::NAT spo11-217* |
| **S96-179** | Same as S96, but *spo11::HYG ura3::NAT spo11-179* |
| **YJMspo11** | *spo11::HYG MATα ho::hisG lys2 cyh* |
