# Supplementary material for: High Throughput Sequencing Reveals Alterations in the Recombination Signatures with Diminishing Spo11 Activity
Source: PLoS Genet. 2013 Oct 31;9(10):e1003932. doi: 10.1371/journal.pgen.1003932 (PMC3814317; doi:10.1371/journal.pgen.1003932)
Supplement: Text S1 — Methods. (PDF) [file pgen.1003932.s011.pdf]

Methods S1

Justification of using log normal distribution.

The distribution from De Massy could be simplified by a gamma-type distribution. From preliminary data fitting of Tract-Seq simulated tracts, a log-gamma distribution was found to be the best fitting gamma-type distribution. Thus, we have compared the means obtained by fitting a log-gamma vs. a log-normal distribution for WT COs, WT NCOs, HI COs, HI NCOs, LO COs, LO NCOs, NCO 2 Chtd and NCO w/CO tracts. They varied on average by 1.25 bp (range=0-5 bp). We next compared the % of prototrophs at *ARG4* as well as the CO association for Arg prototrophs. Both measures did not vary significantly and fit the data about equally well.

To find the best fitting distribution for our data between a log-normal and a log-gamma distribution in a more quantitative way, we computed a measure of the goodness-of-fit for non-nested models, the Deviance Information Criterion (DIC), for each model of the 8 types of tracts mentioned above. The DIC is a quality of fit statistic, similar to the residual sum of squares (RSS), that favors models with a better fit after penalizing for complexity (similar complexity for log-normal vs. log-gamma distributions); thus a small DIC is preferred. In all 8 cases, the DICs for the log-normal distributions were equal or smaller than those for the log-gamma distributions, providing empirical evidence for our choice. However, those DIC differences were minimal and both distributions provide good fits.

Supplemental References

1. Arora C, Kee K, Maleki S, Keeney S (2004) Antiviral protein Ski8 is a direct partner of Spo11 in meiotic DNA break formation, independent of its cytoplasmic role in RNA metabolism. Mol Cell 13: 549–559.

2. de Massy B (2003) Distribution of meiotic recombination sites. Trends Genet 19: 514–522.

3. Kelley LA, Sternberg MJE (2009) Protein structure prediction on the Web: a case study using the Phyre server. Nat Protoc 4: 363–371. doi:10.1038/nprot.2009.2.

4. Nag DK, Pata JD, Sironi M, Flood DR, Hart AM (2006) Both conserved and non-conserved regions of Spo11 are essential for meiotic recombination initiation in yeast. Mol Genet Genomics 276: 313–321. doi:10.1007/s00438-006-0143-7.

5. Rockmill B, Fung JC, Branda SS, Roeder GS (2003) The Sgs1 helicase regulates chromosome synapsis and meiotic crossing over. Curr Biol 13: 1954–1962.

6. Winzeler EA, Richards DR, Conway AR, Goldstein AL, Kalman S, et al. (1998) Direct allelic variation scanning of the yeast genome. Science 281: 1194–1197.
